# Photochemical Synthesis of Gold and Silver Nanoparticles—A Review

**DOI:** 10.3390/molecules26154585

**Published:** 2021-07-29

**Authors:** Nicole Jara, Nataly S. Milán, Ashiqur Rahman, Lynda Mouheb, Daria C. Boffito, Clayton Jeffryes, Si Amar Dahoumane

**Affiliations:** 1 School of Biological Sciences and Engineering, Yachay Tech University, Hacienda San José s/n, San Miguel de Urcuquí 100119, Ecuador; nicole.jara@yachaytech.edu.ec (N.J.); nataly.milan@yachaytech.edu.ec (N.S.M.); 2 Center for Midstream Management and Science, Lamar University, Beaumont, TX 77710, USA; arahman2@lamar.edu (A.R.); cjeffryes@lamar.edu (C.J.); 3 Laboratoire de Recherche de Chimie Appliquée et de Génie Chimique, Hasnaoua I, Université Mouloud Mammeri B.P.17 RP, Tizi-Ouzou 15000, Algeria; lynda.mouheb@ummto.dz; 4 Department of Chemical Engineering, Polytechnique Montréal, C.P. 6079, Succ. Centre-Ville, Montréal, QC H3C 3A7, Canada; daria-camilla.boffito@polymtl.ca; 5 Center for Advances in Water and Air Quality, The Dan F. Smith Department of Chemical Engineering, Lamar University, Beaumont, TX 77710, USA

**Keywords:** photochemistry, nanoparticle synthesis, Au NPs, Ag NPs, green processes, mechanistic aspects, applications

## Abstract

Nanomaterials have supported important technological advances due to their unique properties and their applicability in various fields, such as biomedicine, catalysis, environment, energy, and electronics. This has triggered a tremendous increase in their demand. In turn, materials scientists have sought facile methods to produce nanomaterials of desired features, i.e., morphology, composition, colloidal stability, and surface chemistry, as these determine the targeted application. The advent of photoprocesses has enabled the easy, fast, scalable, and cost- and energy-effective production of metallic nanoparticles of controlled properties without the use of harmful reagents or sophisticated equipment. Herein, we overview the synthesis of gold and silver nanoparticles via photochemical routes. We extensively discuss the effect of varying the experimental parameters, such as the pH, exposure time, and source of irradiation, the use or not of reductants and surfactants, reagents’ nature and concentration, on the outcomes of these noble nanoparticles, namely, their size, shape, and colloidal stability. The hypothetical mechanisms that govern these green processes are discussed whenever available. Finally, we mention their applications and insights for future developments.

## 1. Introduction

Nanoparticles (NPs) have unique chemical and physical properties compared to their bulk counterpart due to their high surface area and outstanding electronic, optical, magnetic, and catalytic properties, to name a few [1,2,3,4]. The production of valuable nanoparticles for targeted applications is currently a fast-growing field of research [1,5,6]. Due to the ever-growing and sustained demand for nanomaterials, new and efficient synthesis methods are devised, including physical, chemical, and biological methods [6,7,8,9].

**Figure 1:**
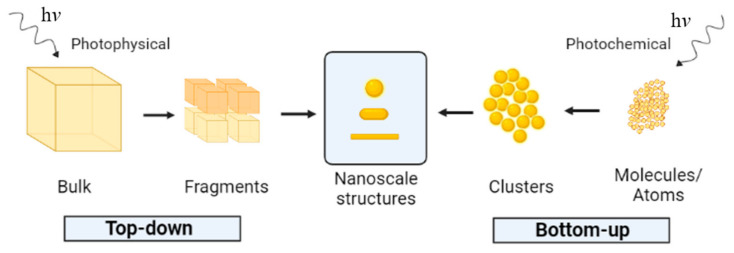
Illustration of the photochemical formation of nanoparticles.

Inorganic NPs are commonly synthesized via three different approaches [10,11,12,13]. Biological methods rely on biomolecules [14,15,16,17], plants [18], fungi [19,20], yeast [21,22,23], algae [24,25,26,27,28], and living cells and organisms [29,30] to promote the synthesis of NPs [4,31,32]. On the other hand, physical methods often involve specialized equipment [33,34,35], while chemical routes usually require numerous chemicals [36,37,38,39]. To avoid using chemical compounds and generating harmful by-products, interest in developing environmentally friendly methods, such as nanoparticle photoinduced synthesis, is increasing [40,41,42,43]. In addition to being easy and simple to implement, the photoinduced method is, in many ways, a green process that relies on biological extracts [44], biomolecules [45,46], and restricted use of chemicals [47] as the reducing agent and/or the stabilizer rather than harmful substrates. Moreover, it diminishes or eliminates the generation of waste and hazardous by-products [42,48,49]. Depending on the starting material, the photoinduced synthesis can be classified as photophysical if a bulk material is downsized to nanomaterials (top–down pathway) or photochemical when atoms and molecules are assembled to build up the nanoparticles (bottom–up pathway) [42,49,50,51,52], as indicated in Figure 1. The photochemical synthesis method has comparative advantages, such as rigorous irradiation control, room temperature operation, simple and inexpensive equipment, and does not require highly skilled personnel [40,53,54,55,56].

Nanoparticle use in various fields has gained steady, growing attention, and metallic nanoparticles are no exception [57,58,59,60]. Metallic nanoparticles have a diameter between 1to a few hundred nanometers [61] and exhibit outstanding electronic, optical [62], and physicochemical [3] properties that are unique from their bulk crystal form [61,63,64].

In the past decades, silver NPs (Ag NPs) and gold NPs (Au NPs) have been extensively studied [65]. They have size- and shape-dependent properties that enable their application in numerous biological and biomedical areas [66,67,68,69], such as biosensing [70], drug delivery [71,72], medical imaging [65], and pharmaceutics [73]. Due to their antimicrobial activity [57], they are also exploited in wound dressing and healing [74].

Au NPs have superior surface plasmon resonance (SPR) properties [11], versatile surface bioconjugation [75,76], high electron conductivity [1], high colloidal stability [65], and low toxicity [72,77]. Likewise, Ag NPs possess unique SPR band [78], optical properties [79,80], good electrical conductivity, chemical stability, and catalytic properties [81] in addition to excellent antibacterial activity [82,83].

Over the years, Au NPs and Ag NPs have gained increasing importance and shown remarkable, versatile bioapplications. This review provides the readership an overview of the principal studies portraying the photochemical synthesis of Au NPs and Ag NPs using ultraviolet and visible light in addition to hypothetical mechanistic aspects, specific applications, and future developments.

## 2. Photochemical Processes

Photochemical processes have gained significant attention in the fabrication of metallic NPs due to the enhanced spatial and temporal control these techniques offer [49,84]. A typical experiment exposes solutions containing the metal precursors to visible or ultraviolet (UV) light. Compared to other methods, such as chemical methods, photochemical routes in nanotechnology are advantageous as they avoid the use of toxic or harmful compounds, do not rely on expensive instrumentation or highly skilled personnel, and, most importantly, can be carried out at ambient conditions, i.e., room temperature and atmospheric pressure [40,54,56].

The fabrication of metallic NPs in solution uses several initial reagents, such as the metal precursor (salt or complex), reducing agent, and sometimes, stabilizing agent [85,86]. The photochemical process begins with the reduction of the metal precursor, from n^+^ valence (M^n+^) to its zero-valence state (M^0^) by the photocatalyzed action of the reducing agent [49,87]. The M^0^ form nucleation centers or nuclei that subsequently grow and aggregate to give rise to metallic NPs [88].

Stabilizers and capping agents are of utmost importance since they are used to control the formation of homogenous metallic NPs of desired size and shape [89,90], prevent their agglomeration, and improve their colloidal stability [91,92,93,94]. Among the capping agents, polymers are excellent for trapping and protecting the NPs against their oxidation and coalescence [66,95]. Moreover, the different chemical structures of the polymers enable specific interactions with the metallic surface, triggering significant variations in the size and shape of the obtained metallic NPs [66]. Some studies have detailed the impact of various polymers on the characteristics of Ag NPs and Au NPs, such as poly(vinyl pyrrolidone) (PVP) [2,96,97], polymethacrylate (PMA) [98], poly-, mono-saccharides and proteins (chitosan, glucose, dextrose, gelatin) [41,89,94,96], sodium dodecyl sulfate (SDS) [99], and natural rubber (NR) latex [66]. Additionally, the experimental parameters determine the NP features and morphology, namely, the use and nature of the stabilizers, pH of the reaction medium, precursor concentration, exposure time, and, importantly, light source and wavelength [100,101].

## 3. Synthesis of Gold and Silver Nanoparticles under UV Light Irradiation

Ultraviolet radiation is located between visible light and ionizing radiation. Its wavelength ranges from 100 nm to 400 nm and can be divided into three groups: UV-A (315–400 nm), UV-B (290–315 nm), and UV-C (100–290 nm) [2,102]. As the energy is inversely proportional to the wavelength, UV-C radiation possesses the highest energy and can cause irreversible cell damage [102].

The use of a photosensitizer enhances light absorption by the reaction medium. UV light directed to photosensitizers in appropriate solvents generates radicals that are involved in the M^n+^ reduction process and subsequent formation of metallic NPs. The ketone family is, by far, the most frequently used photosensitizers in the synthesis of metallic NPs [49]. Under UV irradiation, ketones form ketyl radicals that act as reducing species. For instance, benzoin is a ketone that produces ketyl radicals by photolysis. A Norrish type 1 cut (α-cleavage) occurs in the benzoin, generating short-lived triplet ketyl radicals that concomitantly trigger the reduction of cationic noble metal precursor because of radical quenching. This efficiently converts metal ions into metallic NPs [7,103]. UV irradiation sources are mercury lamps and UV light emitting diode (LED) mounted in photoreactors [95]. Under UV irradiation, the experimental parameters, such as the pH solution, illumination time, and reagents’ concentration, impact the size and the shape of synthesized metallic NPs [91,104]. For example, Gabriel et al. performed the synthesis of spherical Ag NPs on a chitosan/clay nanocomposite, using different concentrations of chitosan as the stabilizing agent [41]. At a low clay content, NPs of a more uniform size (2.7–6.3 nm) are obtained, while, at higher ones, aggregates and NPs of wider size distribution (2–20 nm) are obtained, but are formed faster.

### 3.1. Gold Nanoparticles (Au NPs)

The UV photochemical synthesis of Au NPs is characterized by: (1) the use of light-absorbing solutes and reagents is not always necessary, (2) the reduction of metal ions may occur without reducing agents, and (3) the photon-mediated generation of reducing equivalents is usually well-defined, giving a constant and predictable reduction rate. Furthermore, it does not require sophisticated instruments, making it a practical and low-cost method. Only a UV lamp is needed [47].

#### 3.1.1. Influence of pH

**Figure 2:**
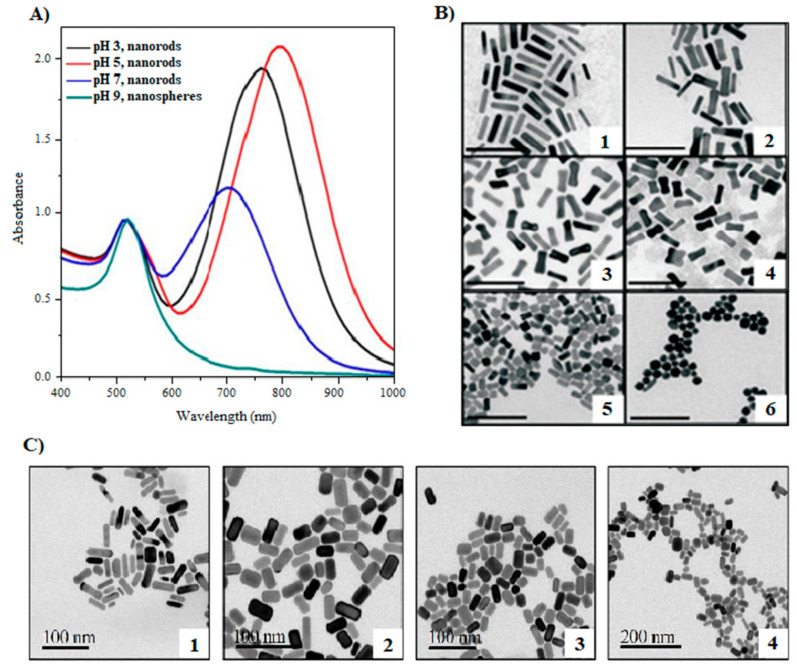
Effect of pH on the formation of Au NPs. (**A**) Absorption spectra of Au NPs synthesized using a 256 nm UV lamp and CTAB as the surfactant at different pH values for 1 h. Adapted from [107]. Published by IOP Publishing under Creative Commons Attribution 3.0 License. (**B**) TEM micrographs of Au NPs formed at different pH values: (1) 1.23, (2) 2.00, (3) 3.00, (4) 4.00, (5) 9.00, and (6) 10.28 (scale bar: 100 nm). Adapted from [108] with permission from Wiley. (**C**) TEM micrographs of Au NPs obtained using a UV lamp (256 nm) and CTAB at different HAuCl_4_ concentrations: (1) 3.0 mM, (2) 4.5 mM, (3) 6.0 mM, and (4) 7.5 mM. Adapted from [110]. Published by The Italian Association of Chemical Engineering under Open Access Policy.

Au NPs can be isotropic (nanospheres) or anisotropic (nanorods, nanowires, nanoshells, etc.). Au nanospheres are characterized by the presence of a single SPR band whose maximum is located at ~520 nm while their nanorod counterpart exhibits two SPR absorption bands: the transversal mode (~520 nm) and the longitudinal mode whose position depends on their aspect ratio, that is, the ratio between their length to their width [46,105,106,107]. During the synthesis of Au NPs, the morphology is primarily controlled by the pH of the reaction medium, precursor concentration, surfactant type, and irradiation time [46,107]. To evaluate the pH influence on the formation of Au NPs, Rodriguez et al. used a UV lamp (256 nm) at various pH values and many reagents, such as the precursor (tetrachloroauric acid, HAuCl_4_; silver nitrate, AgNO_3_), cationic surfactant (hexadecyltrimethylammonium bromide, CTAB), acids (ascorbic and hydrochloric acids), base (ammonium hydroxide), acetone, and cyclohexane [107]. As a result, Au nanospheres predominate in basic solutions (pH = 9) and their UV-Vis spectrum presents one SPR band (515 nm). However, Au nanorods that display two SPR bands (Figure 2A) form in acidic solutions (pH = 3 or 5). Similar observations were made by Cheng et al. [108]. Au nanorods are formed at low pH, while the NPs become more spherical as the pH increases (Figure 2B). Following a similar methodology, Unal et al. screened the pH effect on the Au NP size using red cabbage extract, a renewable feedstock, as both the stabilizer and reducing agent. A pH of 2.5 yielded aggregated NPs of 5–70 nm in size while a pH of 11 produced stable, uniform NPs of 18–30 nm in size [109]. This is mainly due to the rapid nucleation occurring at basic pH yielding the isotropic growth of smaller nanostructures, while the acidic pH causes the nuclei to grow anisotropically [107]. These results highlight the importance of the pH reaction medium in controlling the NP size and shape.

#### 3.1.2. Influence of Precursor Concentration

Tetrachloroauric acid (HAuCl_4_) is the most used gold precursor [84]. The shape of Au NPs is sensitive to the precursor concentration; in addition, the presence of silver nitrate favors the formation of anisotropic NPs since silver ions are directly related to the appearance of the longitudinal SPR band. For instance, Sanabria-Cala et al. studied the influence of HAuCl_4_ and AgNO_3_ concentrations on Au NP morphologies using a 256 nm wavelength UV lamp and the cationic surfactant CTAB [110]. The results highlighted that (i) an increase in HAuCl_4_ concentration decreases the growth of CTAB micelles and yields spherical nanoparticles (Figure 2C) and (ii) an increase in the concentration of silver nitrate supports the formation of anisotropic Au nanorods. Moreover, the nanoparticles tend to form nanospheres in excess of HAuCl_4_ since there is not enough CTAB to form elongated micelles to induce the formation of nanorods [107].

**Figure 3:**
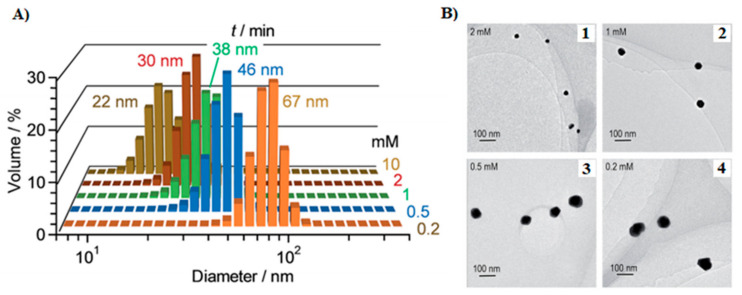
(**A**) Hydrodynamic diameter of Au NPs obtained under 254 nm UV irradiation in the presence of citric acid, at different concentrations, as the reductant and stabilizing agent. (**B**) TEM of Au NPs obtained under xenon lamp (254 nm, 150 mW m^−2^) irradiation for 100 min and different concentrations of citric acid: (1) 2.0 mM, (2) 1.0 mM, (3) 0.5 mM, and (4) 0.2 mM. In the presence of higher concentrations of citric acid, very stable Au NPs with small size are obtained. Adapted from [111]. Published by The Royal Society of Chemistry under Creative Commons Attribution 3.0 Unported License.

Many photochemical methods require reducing and stabilizing agents, such as citric acid, Triton x-100, dendrimers, trisodium citrate, and PVP, among others [111,112]. For instance, Shiraishi et al. studied the influence of the citric acid concentration on the size of Au NPs formed under UV irradiation (254 nm) [111]. Increasing citric acid concentration increases the Au NP formation yield as depicted by the intensity of the SPR band, located at ~530 nm, that goes up, indicating a fast nucleation process and, consequently, the formation of tiny Au NPs (Figure 3). On the other hand, UV irradiation can also be used as the sole reducing agent without any chemical or biological reductants. To that aim, Teixeira et al. relied on 254-nm UV radiation as the light source and polyethyleneimine (PEI) as the stabilizing agent [113]. Besides a stabilizing-free experiment (the control), the concentrations of HAuCl_4_ (precursor) and PEI were varied ([PEI]:[HAuCl_4_] of 5:1, 10:1, and 20:1) to study their impact on the NP size. Similar to the results discussed by Shiraishi et al. [111], as the stabilizer to precursor concentration ratio increases, smaller spherical Au NPs are formed. Moreover, no NPs were obtained in the absence of PEI as this reagent plays an important role in the nucleation step during the NP formation.

Surfactants, which also act as stabilizing agents, play a critical role in the synthesis of Au NPs because they template and control their growth and prevent their shape and size from evolving [88,114,115]. There are several types of surfactants, such as the anionic ones (SDS, sodium dodecylbenzene sulfonate (SDBS), etc.), the cationic ones (dodecyltrimethylammonium bromide (DTAB), cetyltrimethylammonium bromide (CTAB), alkyltrimethylammonium bromide, etc.), and the non-ionic analogs (Triton X-100, Tween X-80, hydrogenated castor oil (HCO), etc.) [114,116]. The surfactant nature and structure affect the NP size. For example, Shang et al. produced Au NPs from HAuCl_4_ and several surfactants [117]. Their results demonstrate that the NP size can be tuned by using different surfactants. Indeed, cationic surfactants favor the formation of large-sized Au NPs owing to their positively charged head that attracts the dissociated AuCl_4_^−^ ions, whereas anionic surfactants produce smaller Au NPs, as their negatively charged head repels the dissociated AuCl_4_^−^ ions, allowing only reduced Au atoms to enter the micelles, resulting in smaller Au NPs.

#### 3.1.3. Greener Alternatives

Since many reductants and stabilizers are toxic and harmful to human health and the environment, eco-friendly compounds constitute a viable alternative in the fabrication of nanomaterials. This may consist, for instance, in using extracts from plant parts (fruits, stems, leaves, etc.) as the reducing and stabilizing agents [109,118,119]. Yulizar et al. used *Polyscias scutellaria* leaf extract under UV irradiation for 2 h to synthesize stable Au NPs of 5–20 nm in diameter [119]. Additionally, these NPs exhibited interesting catalytic activity in the reduction of methylene blue (MB) in the presence of sodium borohydride (NaBH_4_). Similarly, Babu et al. reported the synthesis of Au NPs using the ethanolic extract of *Bacopa monnieri* leaves (BLE) under UV irradiation for 15 min [120]. Compared to the heating route, this method formed Au NPs from 1 mM of HAuCl_4_ with 4% of BLE (400 μg) and was much faster: 15 min using UV light vs. 80 min by heating. Furthermore, UV light produced smaller Au NPs (11 nm) than by heating (21 nm).

### 3.2. Silver Nanoparticles (Ag NPs)

The UV-mediated photochemical synthesis of Ag NPs is a well-established method. It has three main features that fit the principles of Green Chemistry: (i) water is usually the solvent, (ii) environmentally benign reducing agents are used, and (iii) non-toxic capping agents ensure NP stability [121]. Most often, the experiments are carried out at room temperature and atmospheric pressure using common lab equipment. In most cases, silver nitrate (AgNO_3_) is used as the precursor [122,123,124], while polymers and green materials are used as stabilizers and plant extracts [118] as reducing agents.

#### 3.2.1. Influence of pH

The pH of the reaction medium plays an important role in controlling the size of Ag NPs [125,126]. Thus far, the formation of Ag NPs under UV irradiation has been limited to a narrow pH range [125]. Some studies suggest that the Ag NP size tends to decrease in alkaline media due to the rapid nucleation, while acidic media results in larger Ag NPs since the reaction rate is slower [127,128]. Furthermore, the pH affects the absorption of light and the position of the localized SPR (LSPR) band [126,129]. Babusca et al. synthesized Ag NPs via a two-step process: the aqueous mixture of AgNO_3_ and trisodium citrate was heated then irradiated using a UV-C lamp (30 W) for 45 min [126]. The two-step process promotes the efficient synthesis of Ag NPs since the reduction of silver ions is totally completed during the first step. As a result, the pH slightly increased from 5 to 6 while the SPR band maximum blue-shifted from 425 nm to 418 nm, suggesting the formation of smaller Ag NPs; this corroborates studies that reported the influence of increasing pH on the narrowing and position of the Ag NP SPR band [129,130].

#### 3.2.2. Influence of Reducing Agents

**Figure 4:**
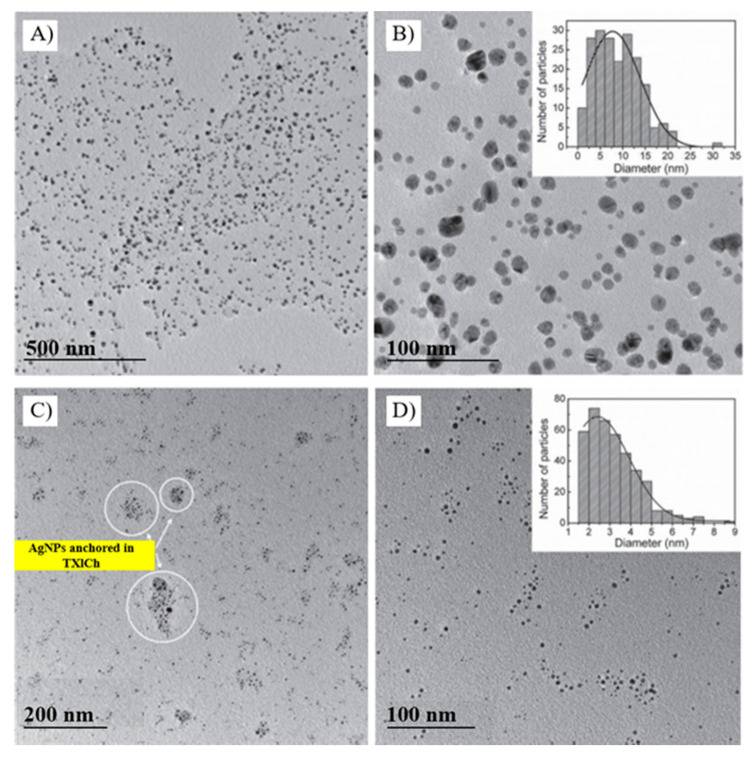
TEM images of spherical Ag NPs/TXICh (as the photoinitiator and stabilizer) in the absence of TEOH (**A**,**B**) and in its presence (**C**,**D**). The reaction mixtures were irradiated using a 365 nm UV light with a power of 92 mW cm^−2^. Adapted from [94]. Published by The Brazilian Chemical Society under Open Access Policy.

Rheima et al. irradiated an aqueous solution of glucose (C_6_H_12_O_6_), used as both the reducing and stabilizing agent, in the presence of the precursor, silver nitrate, with a mercury UV lamp (λ = 365 nm, 125 W) for 30 min to produce highly crystalline hexagonal and spherical Ag NPs with an average size of 20 nm [131]. Besides, Valandro et al. reported the use of other substances, such as 10-oxo-10H-di benzene thiopyran-3-4-dicarboximide chitosan (TXICh) and triethanolamine (TEOH), that act as both the reducing and stabilizing agent [94]. When a UV-LED (λ = 365 nm, 92 mW cm^−2^) irradiated for 4 h an AgNO_3_ solution containing only TXICh, spherical, self-assembled Ag NPs of 2–24 nm in size are obtained (Figure 4A,B). On the other hand, a 15-min irradiation is sufficient to obtain stable, spherical Ag NPs with an average diameter of 2.5 nm when a mixture made of both TXICh and TeOH is used (Figure 4C,D). TEOH is a good hydrogen donor; therefore, it shortens the reduction time by adding more free radicals to the TXLCh-containing reaction medium.

In addition to green reducing agents, such as plant extracts, polymers are also widely used to synthesize Ag NPs under UV irradiation. In the presence of PVP, known to be a good protective agent and NP aggregation inhibitor, Radoń and Łukowiec irradiated AgNO_3_ solution using UV light (λ = 365 nm) for 10 min [132]. When PVP was the reducing agent, the produced Ag NPs were cubes, rods, and spheres with an average size of 50.3 ± 27.5 nm. However, using chloramine T (Cl-T) as the reducing agent at a concentration of 0.25 g L^−1^ resulted in unstable and irregular Ag NPs with a mean size of 11.7 ± 7.2 nm. As Cl-T concentration increases, large and stable Ag NPs are formed. Finally, the Ag NPs ability to reduce methylene blue as a function of the reducing agent was investigated. Cl-T-synthesized Ag NPs were found to be the best catalyst with a first-order rate constant over catalyst mass (K = k/m) of 8.34 g^−1^ s^−1^ vs. 6.3 g^−1^ s^−1^ for PVP.

Various studies showed that a reducing agent is not always required for the UV-mediated synthesis of metallic NPs. For instance, Huang and Yang used only the precursor (AgNO_3_) and aqueous laponite as the colloidal stabilizer [133]. This solution was irradiated by 0.362 mW cm^−2^ of UV light for 1, 3, 18, 46, and 93 h. In the case of 3 h UV irradiation, the obtained Ag NPs were 60–110 nm nanoprisms and pentagons. The 18 h UV exposure resulted in no nanoprisms and significantly decreased mean nanocrystal size when compared to the 3 h UV irradiation. The shortest irradiation time (1 h) yielded larger Ag NPs that lacked colloidal stability.

**Figure 5:**
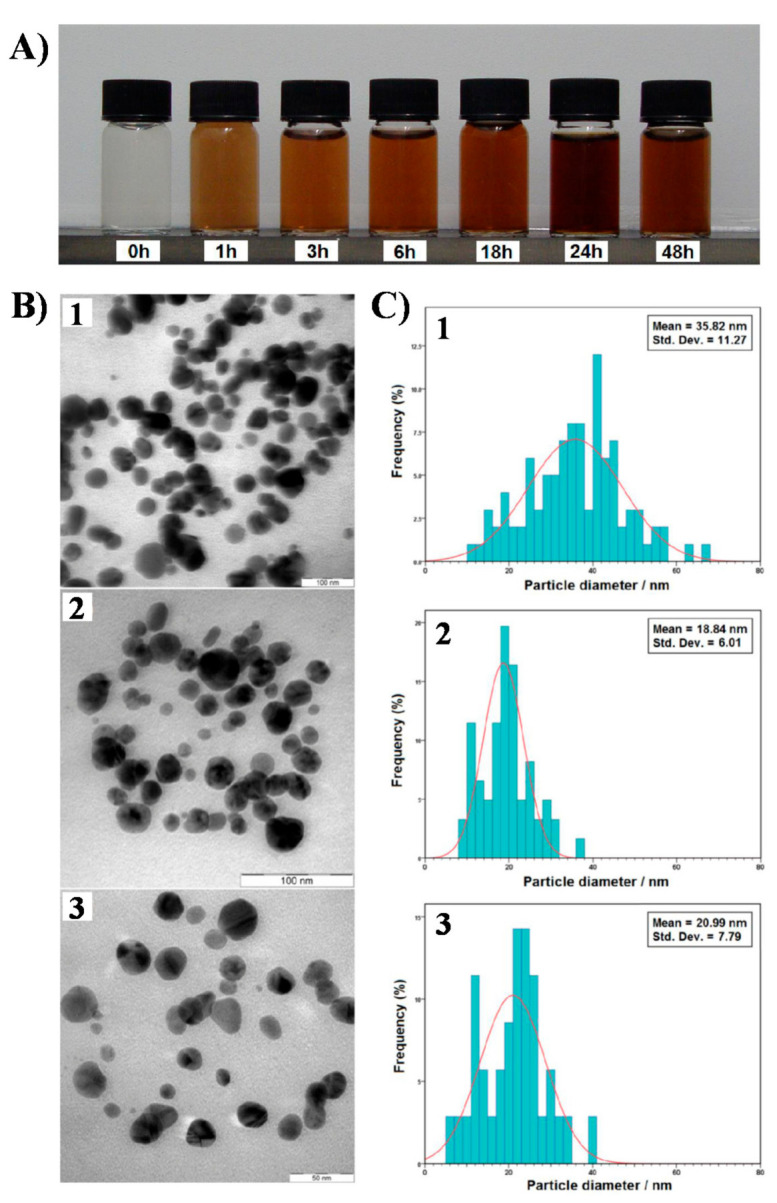
(**A**) Visual aspect of colloidal Ag NPs synthesized in 1% gelatin solution as the stabilizing agent at different UV-A light (6 W) irradiation times. (**B**) TEM images of Ag NPs at different UV irradiation times: (1) 6 h, (2) 24 h, and (3) 48 h. (**C**) Size distribution of Ag NPs obtained at different irradiation times: (1) 6 h, (2) 24 h, and (3) 48 h. Adapted from [89]. Published by MDPI under Creative Commons Attribution License.

Darroudi et al. used gelatin as the green stabilizer with AgNO_3_ as the precursor under UV-irradiation for 48 h [89]. The color changed from yellow to dark brown, depicting the Ag NP formation as the irradiation time increased (Figure 5A). The Ag NPs were analyzed by TEM and UV-Vis spectroscopy. TEM images show a mean Ag NP size of 35.82 nm at 6 h irradiation, 18.84 nm at 24 h, and 20.99 nm at 48 h (Figure 5B). As UV irradiation time increases, the NP size decreases. Furthermore, UV-Vis spectroscopy, used to study the NP colloidal stability, shows that 3 months after their synthesis, the Ag NPs had a slight SPR red shift and absorbance decrease, highlighting their long-term stability (Figure 5C).

### 3.3. Bimetallic Silver–Gold Nanoparticles

Over the last 2 decades, the photochemical synthesis of bimetallic silver–gold NPs has witnessed limited developments when compared to their monometallic counterparts. A few recent works rely on visible light [134,135], but the very vast majority of designed protocols use UV irradiation to produce bimetallic Ag–Au NPs. The photochemically produced mono-metallic NPs may serve as a template, therefore forming the core, for the growth of the other metal, forming the shell. The shell formation might happen under light (e.g., UV) irradiation [136] or simply owing to galvanic exchange [134]. These bimetallic NPs may form spheres [137], decahedrons [134], mixed nanostructures [138], and nanodendrites [139]. In terms of composition, these NPs can consist of alloys [137,140], nanotwins [137,141], core-shell structures [136,142,143,144,145,146,147], and hollow NPs [148].

Mallik et al. were most likely the first to describe, in 2001, the photochemical synthesis of spherical, bimetallic Au_core_–Ag_shell_ NPs via a seed-mediated procedure under UV irradiation [88]. More recently, Kazancioglu et al. reported the fabrication of bimetallic Au–Ag alloy nanoparticles of less than 10 nm under UV irradiation using 2-thioxanthone thioacetic acid-dioxide as a novel photoinitiator; these hybrids possess interesting catalytic activity [140]. This work constitutes a follow-up to a previous study carried out by the same group [53]. After obtaining alginate beads modified with Ag NPs and Au NPs owing to the action of cyclic UV irradiation (10 min ON/10 min OFF for 40 min), Saha et al. achieved the deposition of gold on Ag NPs (Au@Ag NPs) and silver on Au NPs (Ag@Au NPs) [147]. These hybrids were tested for surface-enhanced Raman spectroscopy (SERS) applications. Besides, Korir et al. obtained Au–Ag nanotwins via a two-step photochemical process in the presence of 1% chitosan [141]. First, Au seeds were synthesized starting from chloro(dimethyl sulfide)gold(I) (the precursor) under a medium-pressure mercury vapor lamp (Source 1: 40–48% UV + 40–43% visible) irradiation for 60–90 min or under UVA irradiation for 6 min. Then, AgNO_3_ was added, and the mixture was irradiated using Source 1 for 5–30 min. This resulted in small, isotropic Au–Ag nanotwins of ~30 nm in size. To obtain anisotropic Au–Ag nanotwins bigger than 70 nm, the Au seed solution should be irradiated for 120 min.

Table 1 summarizes the available precursors, reducing agents, and stabilizers used to photochemically synthesize Au NPs and Ag NPs, the NP features (size and shape), and relevant information regarding the pH and UV irradiation (time of exposure, wavelength, power, and source).

## 4. Synthesis of Gold and Silver Nanoparticles under Visible Light Irradiation

Visible light is defined as the radiation that excites the human visual system. Although its spectrum ranges vary, as they depend on the amount of radiant energy that reaches the retina, 360–400 nm is considered to be the lower limit and 760–830 nm to be the upper limit. Visible light is found in greater abundance in the solar spectrum that reaches the Earth’s surface, compared to UV and infrared lights [150,151]. This light can be exploited in the photochemical processes as sunlight, artificial white light, and monochromatic light (e.g., blue light, red light, etc.).

Sunlight is the largest source of renewable and clean energy; it is non-toxic and non-polluting, and does not leave traces in chemical processes [152]. Photo-assisted synthesis is an efficient method to produce NPs, particularly in the case of solar light, which is directly exploited as it is free, non-toxic, environmentally friendly, renewable, shortens reaction time, and produces NPs with controlled and desired characteristics [87,153,154].

### 4.1. Synthesis of Au NPs

Similar to UV-initiated experimental protocols, Ag NPs and Au NPs can be synthesized using sunlight as an inducing light source [155]. For instance, Annadhasan et al. carried out the synthesis of Au NPs using sunlight and N-cholyl-I-valine (NaValC) as both the stabilizing and reducing agent at basic pH (pH = 9) [45]. Au NP formation was completed within ~20 min under sunlight irradiation; a typical SPR band of spherical NPs with a maximum at ~524 nm was recorded. Following a similar methodology, Pienpinijtham et al. synthesized Au NPs of different shapes (triangles, hexagons, and polygons) using starch as the reducing and stabilizing agent under sunlight irradiation for 5 days [156]. A spectrophotometric absorbance at 315 nm, which corresponds to the ion transfer from chlorine to gold in the mixture, was observed within the first day. This band disappeared over the following days and was replaced by a broad SPR band with a maximum absorbance greater than 500 nm, which indicated the formation of Au nanostructures. The same study reported that no nanostructures were formed in the absence of sunlight although, in dark, the reaction lasted 7 days; this highlights the essential role played by this irradiation in forming NPs [156].

### 4.2. Synthesis of Ag NPs

Several manuscripts have described the synthesis of Ag NPs using sunlight. For instance, Tang et al. reported the formation of anisotropic Ag NPs using sunlight [157]. Furthermore, sunlight irradiation has been shown to promote a fast reduction process, resulting in a higher yield when compared to control experiments in the dark. Some procedures relying on sunlight can be performed without the presence of any reducing moieties [6,45,155,158]. SDS is an example of a surfactant used in the synthesis of Ag NPs starting from an AgNO_3_ aqueous solution under sunlight irradiation of ~50.3 mW cm^−2^ intensity for 1 h at room temperature [155]. The Ag NP formation started soon after the experiment was launched; the first color change was observed 5 min after exposure and continued steadily. However, exposure times longer than 5 h did not bring further changes. In comparison, the control experiment in the dark did not yield any color change nor Ag NP formation, highlighting the key role played by sunlight in this process [155,158,159].

### 4.3. Greener Alternatives

The literature details the synthesis of Ag NPs using plant extracts as the reducing agent, such as ginger rhizome extract, and AgNO_3_ as the precursor under sunlight irradiation. Using 5 mL ginger extract (5%) in 95 mL of 1 mM AgNO_3_ under sunlight exposure, a rapid color change from yellow to dark brown occurred within 2 h, indicating the formation of spherical Ag NPs [87]. This holds great promise for the large-scale synthesis of Ag NPs using a free energy source, sunlight, and minimal plant material. Besides, the synthesis of Ag NPs using *Azadirachta indica* leaf extract under 1 h sunlight irradiation and the effect of extract amount on Ag NP features have been reported [160,161]. When the amount of leaf extract was 1, 2, and 3 mL, the size of the synthesized nanocrystals decreased from ~107 to ~89, and ~76 nm, respectively [160]. This trend corroborates what was extensively reviewed in the literature [162]. Mankad et al. showed that extending the irradiation time (5, 10, 15, and 20 min) resulted in faster processes as the color change (from yellow to reddish-brown) intensified, obtaining the smallest NP size (~68 nm) with 5 mL of leaf extract and the longest sunlight exposure [161]. The use of *Piper longum* catkin extract for the synthesis of Ag NPs has attracted increasing attention [163,164,165]. Sunlight irradiation triggers the rapid formation of 15–40 nm Ag NPs [164]. Papaya extract promotes the synthesis of Ag NPs of 35–50 nm in size when exposed to sunlight irradiation for 15 min [166], while pomelo peel extract (PPE) yields Ag NPs of 20–30 nm in diameter when sunlight-irradiated for 30 min [158]. The authors proposed that blue light plays the main role in reducing Ag^+^ ions by inducing the tautomerization of the flavonoids in PPE, releasing the reactive hydrogen atoms responsible for the reduction. The authors also emphasized that using sunlight resulted in faster synthesis than heating.

Other studies shed light on the effects of solar irradiation intensity on Ag NP formation kinetics. For instance, Wei et al. produced Ag NPs when the reaction medium containing AgNO_3_ and *Bacillus amyloliquefaciens* cell-free extracts were subjected to different intensities of solar irradiation (3×, 4×, 5×, and 7× 10^4^ l×) for 100 min [167]. The reduction process was evidenced by the appearance of a light brown color after only 1 min, then yellow, and finally orange–red after 100 min irradiation. The Ag NP formation was confirmed using UV-Vis spectroscopy, which showed an SPR maximum at 423 nm. The obtained Ag NPs were circular and triangular and ~15 nm in size regardless of the light intensity. However, the maximum SPR peak intensity increased when the light intensity increased, highlighting the direct relationship between light intensity and Ag NP formation kinetics and yield.

### 4.4. Impact of Experimental Parameters

**Figure 6:**
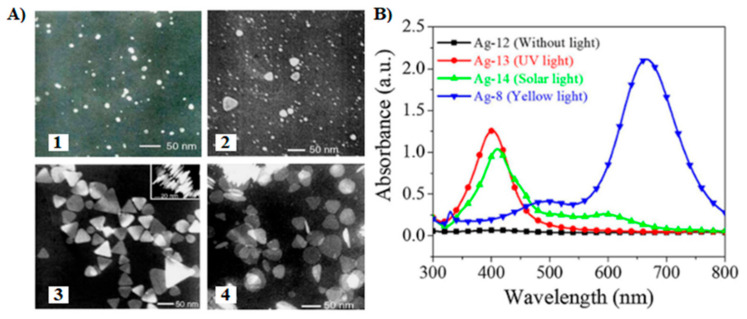
(**A**) Impact of the irradiation time on the shape of Ag NPs obtained from AgNO_3_, sodium citrate, and sodium borohydride irradiated with a sodium lamp (589 nm, 70 W): (1) 0 h, (2) 1 h, (3) 3.5 h, and (4) 5 h. Adapted from [168] with permission from Elsevier. (**B**) UV-Vis absorption spectra of Ag NPs synthesized under different illumination sources for 180 min. The NaBH_4_ was added dropwise to the aqueous solution containing citrate and AgNO_3_; the pH was controlled by adding NaOH. Adapted from [169] with permission from Elsevier.

Jia et al. irradiated an aqueous solution containing AgNO_3_, sodium citrate, and sodium borohydride with a sodium lamp (70 W, 589 nm) [168]. TEM images show the morphology changes. Before irradiation, the NPs were spherical, with a diameter of 2–15 nm. After 1 h, small triangular Ag NPs (T-Ag NPs) were visible and, after 3.5 h, truncated triangular NPs with 40–100 nm in edge length were observed. Finally, at 5 h, the NPs became more truncated and showed a disk-like shape (Figure 6A). Moreover, Nguyen et al. varied the pH and irradiation source during the photochemical synthesis of T-Ag NPs [169]. The results showed that the absorption peaks were between 520 nm and 580 nm at pH 11–12. On the other hand, a peak at 650 nm, characteristic of T-Ag NPs, was observed at pH 9. Besides a dark control, different irradiation sources were used, such as a sodium lamp (λ = 589 nm), a UV-C lamp (λ = 254 nm), and a solar lamp (full spectrum light) for 180 min (Figure 6B). In the dark or under UV light exposure, only ~2 nm and ~6 nm Ag NPs, respectively, were formed. Under solar lamp illumination, several morphologies were obtained with a mean size of ~7 nm, while using the sodium lamp yielded T-Ag NPs of ~28 nm in edge length.

**Figure 7:**
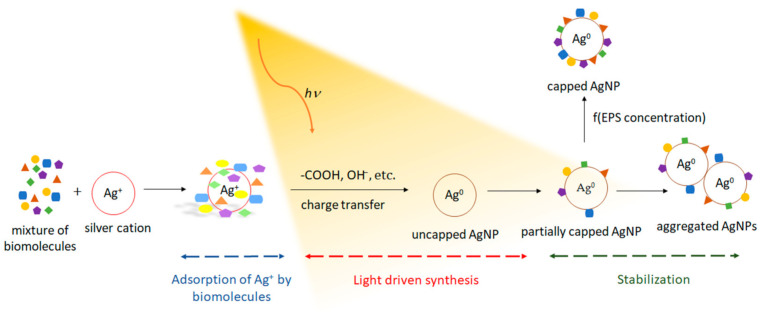
Hypothetical mechanism of the light-driven synthesis of Ag NPs using exopolysaccharides extracted from the green microalga *C. reinhardtii*. Adapted from [17]. Published by MDPI under Creative Commons Attribution (CC BY) License.

Rahman et al. used algal production platforms to synthesize Ag NPs facilitated by visible light [17,30,170]. As a result, visible light has a direct effect on the formation of Ag NPs when mediated by the extracellular polymeric substances (EPS) of the green microalga *Chlamydomonas reinhardtii* [17]. The light-dependent nature of this process was revealed by the acidification of the reaction media that only occurred under light irradiation. Moreover, increasing light intensity induced the pH to further decrease and the Ag NP production rate to increase. This led to a constitutive relationship between photons and Ag NP production rate. Additionally, a hypothetical biosynthesis mechanism based on light-independent adsorption of Ag^+^ by the EPS biomolecules and photocatalytic reduction of Ag^+^ to its metallic counterpart, i.e., Ag^0^, was proposed. It was further established that (i) the EPS could maintain their bioreductive capabilities in the dark; however, photon energy is required for their activation, and (ii) the increase in SPR band intensity of the as-produced Ag NPs is related to the light input, while the final yield is related to the EPS concentration (Figure 7).

Several studies have also explored the biosynthesis of Ag NPs and Au NPs under visible light exposure, especially using the photosynthetic machinery and pigments of living organisms and microbes [8,171,172,173]. For instance, extracted C-phycocyanin pigment from cyanobacteria has been reported to promote the formation of Ag NPs only under light illumination [174]. Additionally, intact chloroplasts harvested from microorganisms and plants have been successfully used to synthesize Ag NPs, Au NPs, and bimetallic Ag–Au alloy NPs; however, they all require light input [175,176]. Shabnam et al. reported that, under light irradiation, photosynthetic electron transport in the thylakoids/chloroplasts reduced Au^3+^ to Au^0^ to promote the formation of Au NPs [175]. The group reported 5–20 nm sized Au NPs formed via a light-driven electron transport mechanism that uses light energy to transport electrons from water molecules to different organelles within the cell [175,176]. While the reductive capacities of chloroplasts are evidently understood, how light input affects the specific roles played by the many other green parts of organisms during the NP formation is yet to be investigated [29].

Table 2 depicts the photochemical synthesis of Ag NPs and Au NPs; it summarizes the available precursors, reducing agents, and stabilizers used, the NP features (size and shape), and relevant information regarding the pH and visible light irradiation (time of exposure, wavelength, power, and source).

## 5. Applications of Photochemically Produced Ag NPs and Au NPs

Metallic NPs have been used in medical applications due to their versatility and unique characteristics, such as biocompatibility, colloidal stability, optical properties, straightforward surface functionalization, among others [181,182]. Due to their large surface area, it is possible to load large amounts of drugs onto NPs to design NP-based drug delivery systems that reduce the risk of side effects, target the desired part of the body, and increase the efficiency of the drug. For instance, Au NPs have been widely used for cancer treatment [181,182]. Similar to their analogs obtained via other approaches, photochemically produced Ag NPs and Au NPs possess useful physico-chemical and biological properties that have enabled them to be applied in countless fields, such as nanomedicine, catalysis, and environment. For instance, Dizman et al. used photochemically synthesized Au NPs to deliver doxorubicin (Dox) to treat leukemia. Using MTT assay, the authors determined that the cytotoxicity of these nanoplatforms, made of Au NPs loaded with 5 μL of Dox, was higher in the case of cancer cells when compared to normal cells, making them a potential candidate for cancer treatment [183]. Similar results were obtained by Licciardi et al. who relied on spherical Au NPs conjugated with inulin folate (INU-FA) and loaded with Dox to treat breast cancer cells [184].

The use of visible and UV light enabled size control during the synthesis of Ag NPs, promoting the formation of small-sized NPs and avoiding the formation of aggregates [41,153]. The small size of Ag NPs makes them more effective against bacteria. The oxidation of silver atoms at the NP surface triggers the release of Ag^+^ ions that interact with bacterial proteins and enzymes, which inactivate and interrupt the cell metabolic processes yielding the cell lysis; this effect becomes even stronger when smaller NPs are used [185]. Ag NPs present high toxicity against some bacteria, such as *Escherichia coli* [20,41] and *B. subtilis* [65]. Therefore, these Ag NPs may be used as nano-coatings in surgical devices and implants and as antibacterial agents in wound-healing bandages.

Surface-enhanced Raman spectroscopy (SERS) is a powerful technique that allows the detection of trace amounts of various substances of interest in different fields, such as medicine, chemical analysis, and environmental safety [186,187]. Noble metallic NPs, especially those made of gold and silver, are among the best substrates for SERS [147]. For example, Xu et al. deposited Au NPs, synthesized via a photochemical process, on the surface of zinc oxide nanorods (ZnO NRs) to form ZnO@Au NRs as a substrate for SERS in the detection of methylene blue to a detection limit as low as 0.8 μg L^−1^, demonstrating their potential to detect organic pollutants in wastewater [188]. Similarly, Zhou et al. used a 6-day sunlight exposure to produce Au nanosheets for SERS applications. Most importantly, they found that ~50 nm is the optimal thickness of Au nanosheets for SERS applications [189].

Numerous reports describe the use of Au NPs and Ag NPs to detect and biosense viruses [190,191], pathogens [192], DNA [193,194], pesticides [187], and toxins [195]. Taking advantage of the good electrochemical activity of Au NPs combined with the high specific surface area of ZnO NRs, Xu et al. relied on the photochemical synthesis to develop a sensor of densely deposited Au NPs on the surface of ZnO NRs to detect nitric oxide (NO) released by human umbilical vein endothelial cells (HUVEC) [196]. For environmental detection, Çinko et al. designed, via a photochemical process, Au NPs/polymer nanocomposite films capable of detecting benzene, toluene, and xylene vapors [197]. Besides, water-stable, blue-fluorescent silver nanoclusters (Ag NCs) are used in the stable and sensitive detection of hydrogen peroxide (H_2_O_2_) [198]. To prepare this fluorescent probe, glutathione (GSH), used as a capping agent, reacted with Ag^+^ under UV irradiation to yield Ag NCs. As a result, a response time of 2 s is achieved in the detection of H_2_O_2_, whose monitoring is of utmost importance in biological and chemical systems. Moreover, Ag NPs produced under sunlight irradiation can be used for the colorimetric detection of Hg^2+^ with a linear range of 50 nM–500 µM, where a color change of the solution from dark brown to transparent was observed [199]. In addition, the SPR band intensity confirms the high selectivity and sensitivity of these Ag NPs for Hg^2+^ detection.

## 6. Conclusions and Future Developments

The applicability of Au NPs and Ag NPs in different areas, such as industry, medicine, and agriculture, has triggered a great interest for researchers to devise facile and innovative methodologies for controlled, reproducible, and scalable synthesis with acute environmental concerns. In this context, light-driven routes are of choice as they take advantage of UV and visible light to provide a clean and convenient method for the efficient and rapid synthesis of Au NPs and Ag NPs. In this article, we systematically reviewed the key parameters that materials scientists have considered in implementing their experiments towards the production of these noble metal NPs. As a consequence, reagent concentrations, type of light (natural vs. artificial, UV vs. visible, polychromatic vs. monochromatic), irradiation time, the use or lack of reducing/stabilizing agents, the reaction mixture pH, etc. are crucial parameters that greatly impact the characteristics of the obtained NPs, namely, their size, shape, and colloidal stability; the latter affecting greatly the properties (optical, catalytic, biological) that determine their bio-applications, such as biocidal activity, biosensing, drug delivery nanoplatforms, and catalysis, to name the most commonly reported applications in the literature.

Future work should focus on the appropriate design of experiments to investigate the effect of varying several experimental parameters on the obtained NPs. To that end, scientists may rely on well-established designs of experiments (DoE) to produce NPs of desired compositions (e.g., metallic, oxides, chalcogenides, alloys) and features. Moreover, further surface functionalization of these photochemically obtained NPs should be investigated as a milestone towards their emerging, fast-growing, and diversifying bio-applications. Finally, the scalability of these photoprocesses using photobioreactors, such as the ones used in culturing microalgae, should be thoroughly examined, as this is another key criterion in the economic viability and cost-effectiveness of these green NP production routes.

## Figures and Tables

**Table 1:** UV-driven photochemical synthesis of Au NPs and Ag NPs.

Metal	Precursor	Reducing Agent	Stabilizer/ Surfactant	Irradiation Source, Wavelength and Power	Exposure Time	pH	Size (nm)	Shape	Ref.
Au NPs	HAuCl_4_	-	-	6 W, λ = 365 nm	1 h	-	-	Nanorods	[149]
Au NPs	HAuCl_4_	Sodium dodecyl benzene sulfonate (SDBS)	Id. *	300 W high-pressure mercury lamp	12 h	-	3–4	Spherical NPs	[117]
Au NPs	HAuCl_4_	Sodium dodecyl sulfate (SDS)	Id.	300 W high-pressure mercury lamp	12 h	-	4–5	Spherical NPs	[117]
Au NPs	HAuCl_4_	Extract of cornelian cherry	Id.	6 W UV lamp (365 nm)	2 h	-	19	Spherical NPs	[118]
Ag NPs	AgNO_3_	Extract of cornelian cherry	Id.	6 W UV lamp (365 nm)	2.5 h	-	16	Spherical NPs	[118]
Au NPs	HAuCl_4_	Leaf extract of *Polycias scutellaria*	Id.	UV lamp	2 h	-	5–20	Spherical NPs	[119]
Au NPs	HAuCl_4_	Extract of red cabbage	Id.	6 W power UV (365 nm)	20 min	7	25	Spherical NPs	[109]
Au NPs	HAuCl_4_	Ethanolic leaf extract of *Bacopa monnieri*	Id.	UV lamp (254 nm)	15 min	-	11	Spherical NPs	[120]
Ag NPs	AgNO_3_	Poly(methacrylic acid) (PMA)	Id.	8 W UV lamp (365 nm)	1 h	4	8	Spherical NPs	[98]
Ag NPs	AgNO_3_	Chitosan	Id.	UV-LED (365 nm)	15 min	-	30	Spherical NPs	[94]
Ag NPs	AgNO_3_	PMA	Id.	6 W UV lamp and 25 W UV lamp	1 h	9	10	Spherical NPs	[123]
Ag NPs	AgNO_3_	-	Laponite aqueous suspension	UV light 0.362 mW cm^−2^	3 h	-	60 110	Mainly nanoprisms and pentagons	[133]
Ag NPs	AgNO_3_	Glucose	Id.	UV light (λ = 365 nm; 125 W)	30 min	-	20	Hexagonal and spherical NPs	[131]
Ag NPs	AgNO_3_	Thiopyran-3-4-dicarboximide (TXICh)	Id.	UV LED (λ = 365 nm; 92 mW cm^−2^)	4 h	-	2–24	Self-assembled spherical NPs	[94]
		TXLCh/Triethanolamine (TEOH)			15 min	-	2.5	Stable spherical NPs	
Ag NPs	AgNO_3_	-	Gelatin	UV reactor (UV-A, 6 W)	24 h	-	19	-	[89]
Ag NPs	AgNO_3_	Poly(vinyl pyrrolidone) (PVP)	-	UV light (λ = 365 nm)	10 min	-	11.7 ± 7.2	Cubes, rods and spheres	[132]
Ag NPs	AgNO_3_	-	Poly(acrylic acid) (PAA)	Low-pressure mercury lamp (λ = 253.7 nm)	1 h	-	30–50 nm in width	Nanofilaments	[138]
Au NPs	HAuCl_4_						10–30	NPs	

* When the stabilizer is the same as the reducing agent.

**Table 2:** Visible light-driven photochemical synthesis of Au NPs and Ag NPs.

Metal	Precursor	Reducing Agent	Stabilizer/Surfactant	Irradiation Source	Exposure Time	pH	Size (nm)	Shape	Ref.
Ag NPs	AgNO_3_	-	Sodium dodecyl sulfate (SDS)	Sunlight (50.3 mW cm^−2^)	1 h	-	5–10	-	[155]
Ag NPs	AgNO_3_	*Zingiber officinale* extract	-	Sunlight	2–20 h	-	4–15	Spherical NPs	[87]
Ag NPs	AgNO_3_	Sodium citrate and sodium borohydride	-	Sodium lamp (70 W; λ = 589 nm)	1 h	-	40–110	Truncated triangular NPs	[168]
Ag NPs	AgNO_3_	-	Cell-free extract of *Bacillus amyloliquefaciens*	Sunlight	100 min	7.2	14.6	Circular and triangular NPs	[167]
Ag NPs	AgNO_3_	*Piper longum* extract	-	Sunlight	-	-	15–40	Monodisperse spherical NPs	[164]
Ag NPs	AgNO_3_	*Carica Papaya* extract	-	Sunlight	15 min	4.5	35–50	-	[166]
Ag NPs	AgNO_3_	*Azadirachta indica* leaf extract	-	Sunlight	5 min	-	67.94	-	[161]
Ag NPs	AgNO_3_	*A. indica* leaf extract	-	Sunlight	60 min	10	75.87–185	-	[160]
Ag NPs	AgNO_3_	*Polygonatum graminifolium* leaf extract	Id. *	Sunlight	30 min	-	3–15	Spherical, circular, and triangular NPs	[177]
Ag NPs	AgNO_3_	Pomelo peel extract	-	Sunlight	30 min	3.5	20–30	-	[158]
Ag NPs	AgNO_3_	*Albizia lebbeck* extract/ Citrate + *A. lebbeck* extract	-	Sunlight (~788 lux)	60 min	-	10–20	Spheroidal NPs	[178]
Ag NPs	AgNO_3_	Sodium citrate	-	Sunlight (~788 lux)	60 min	-	-	Large triangular and hexagonal nanoprisms; small spheroidal NPs	[178]
Ag NPs	AgNO_3_	*Sida retusa* extract	Id.	Sunlight	30 min	-	20–40	Spherical	[179]
Ag NPs	AgNO_3_	*Amentotaxus assamica*	-	Sunlight	32 min	-	39.41	Spherical	[180]
Ag NPs	AgNO_3_	-	Ferredoxin-NADP^+^ reductase and ferredoxin (FNR/FD)	Sunlight	150 min	8	10–15	Spherical	[6]
Ag NPs	AgNO_3_	*Pleurotus citrinopileatus* extract	Id.	Sunlight	180 min	-	7.08 ± 2.92	-	[153]
Ag NPs	AgNO_3_	*P. citrinopileatus* extract	Id.	Blue sunlight	180 min	-	3.18 ± 0.72	-	[153]
Au NPs	HAuCl_4_	*-*	Poly(vinyl pyrrolidone)	Xenon flash lamp	20 ms	-	25.3 ± 11.0	Spherical	[135]

* When the stabilizer is the same as the reducing agent.

## Data Availability

Not applicable.
